# Efficacy of quality improvement and patient safety workshops for students: a pilot study

**DOI:** 10.1186/s12909-020-1982-3

**Published:** 2020-04-23

**Authors:** Kevin P. Shah, Shreya Goyal, Vignesh Ramachandran, Jaden R. Kohn, Jonathan A. Go, Zachary Wiley, Anoosha Moturu, Meera K. Namireddy, Anjali Kumar, Ryan C. Jacobs, Matthew Stampfl, Jesal R. Shah, Justin Fu, Weijie V. Lin, Brandon Ho, Grace Wey, Sophie Y. Lin, Andrew C. Caruso, Lindsey Jordan Gay, Diana E. Stewart, Sara Andrabi

**Affiliations:** 1 Office of Undergraduate Medical Education, Baylor College of Medicine, Houston, TX USA; 2 Department of Medicine, Section of General Internal Medicine, Baylor College of Medicine, Houston, TX USA; 3 Department of Pediatrics, Section of Pediatric Hospital Medicine, Baylor College of Medicine, Houston, TX USA; 4 Department of Emergency Medicine, Baylor College of Medicine, Houston, TX USA

**Keywords:** Quality improvement, Patient safety, Curriculum, Workshops, Medical education, Medical students, Health professions students, Experiential learning, Hands-on, Peer-to-peer teaching, Student-led

## Abstract

**Background:**

While the Association of American Medical Colleges encourages medical schools to incorporate quality improvement and patient safety (QI/PS) into their curriculum, medical students continue to have limited QI/PS exposure. To prepare medical students for careers that involve QI/PS, the Institute for Healthcare Improvement chapter at an allopathic medical school and school of allied health professions initiated self-directed learning by offering student-led workshops to equip learners with skills to improve the quality and safety of healthcare processes.

**Methods:**

In this prospective cohort study, workshops were hosted for medical students between 2015 and 2018 on five QI/PS topics: Process Mapping, Root-Cause Analysis (RCA), Plan-Do-Study-Act (PDSA) Cycles, Evidence Based Medicine (EBM), and Patient Handoffs. Each workshop included a hands-on component to engage learners in practical applications of QI/PS skills in their careers. Change in knowledge, attitudes, and behaviors was assessed via pre- and post-surveys using 5-point Likert scales, and analyzed using either the McNemar test or non-parametric Wilcoxon signed-rank test. Surveys also gathered qualitative feedback regarding strengths, future areas for improvement, and reasons for attending the workshops.

**Results:**

Data was collected from 88.5% of learners (*n* = 185/209); 19.5% of learners reported prior formal instruction in these topics. Statistically significant improvements in learners’ confidence were observed for each workshop. Additionally, after attending workshops, learners felt comfortable teaching the learned QI/PS skill to colleagues (mean pre/post difference 1.96, *p* < 0.0001, *n* = 139) and were more likely to pursue QI/PS projects in their careers (mean pre/post difference 0.45, p < 0.0001, n = 139). Lastly, learners demonstrated a statistically significant increase in knowledge in four out of five skills workshop topics.

**Conclusion:**

Few medical students have formal instruction in QI/PS tools. This pilot study highlights advantages of incorporating an innovative, student-directed modified ‘flipped classroom’ methodology, with a focus on active experiential learning and minimal didactic instruction.

## Background

Although progress has been made towards designing safer healthcare systems, there remains much work to be done. Further engagement from healthcare providers and trainees is critical to mitigate preventable adverse events and maximize therapeutic outcomes for patients. A gap between theoretical frameworks of improvement science and their practical application is readily apparent [1, 2]. Based on recent healthcare trends, the majority of published quality improvement and patient safety (QI/PS) data continues to be centered on clinical outcomes and improvement of care processes [3–5]. Less data is readily available regarding QI/PS medical education, especially in undergraduate medical education.

Previous reports demonstrate that practicing clinicians are able to effectively learn QI/PS with various teaching and learning methods [6]. As QI/PS initiatives continue to expand, it is both logical and essential for trainees to learn about basic principles early in training in order to raise awareness, prevent medical errors and ultimately cultivate a culture of safety [7–10]. Medical students should have the aptitude to recognize safety concerns, assist in improving care processes, understand systems-based strategies for improvement, apply evidence-based medicine to practice, and communicate effectively with both colleagues and patients regarding errors or near misses [11, 12].

The World Health Organization (WHO) and the Association of American Medical Colleges (AAMC) encourage medical schools to incorporate QI/PS into curriculum taught to all medical students [13, 14]. However, studies show that medical students continue to have limited or inadequate QI/PS exposure and knowledge [15–17]. Large variation exists within methodology of teaching QI/PS to students in terms of types of skills taught, amount of material covered, the stage of training at which medical students are taught QI/PS, and the teaching strategy employed (didactic vs. experiential hands-on learning) [11, 15, 18, 19].

To address gaps in curriculum and improve overall quality of QI/PS education, we designed an interventional pilot study to assess the impact of self-directed educational QI/PS workshops for students. The objectives of this initiative were to: (1) develop student-led, standardized curriculum for basic QI/PS skills, (2) design an innovative hands-no approach to teaching QI/PS to learners, and (3) assess changes in learners’ knowledge, attitudes, and behaviors regarding QI/PS.

## Methods

### Study design

The prospective cohort study was conducted by student leaders within the Institute for Healthcare Improvement (IHI) Open School Chapter at an institution with an allopathic medical school and a school of allied health professions. The IHI Open School Chapter hosted educational hands-on workshops on five QI/PS skills from 2015 to 2018. Workshops were held during lunch or evening hours and attendance was voluntary for both medical and physician assistant (PA) students. Topics taught included patient handoffs, root-cause analysis (RCA), process mapping, evidence-based medicine (EBM), and plan-do-study-act (PDSA) cycles. All workshops were primarily student-generated or student-led through peer-to-peer teaching, with assistance and support from fellows and faculty. Curriculum was reviewed by subject-matter expert faculty prior to the workshops. Data was collected in the form of pre- and post-workshop surveys administered prospectively to all student attendees. Completing the survey was voluntary.

In addition to these workshops, QI/PS education during medical training at our institution consisted of a mandatory Patient Safety course during the second year of medical school as well as optional preclinical electives offered by the IHI Open School Chapter.

### Workshop structure

All workshops were designed to include both a didactic component and a hands-on interactive component. A methodology of teaching, in which students focus on active learning during each session rather than didactic learning, was utilized [20, 21]. This ‘modified’ flipped classroom methodology leveraged the core principle of focus on application of content rather than attainment. However, it was considered to be modified primarily because students did not receive resources or materials to review prior to attending the workshops. Each workshop had a variation of the following basic structure:

1. Completion of pre-workshop survey
2. Brief didactic session to teach general principles of QI/PS in addition to information pertinent to learning a specific QI/PS skill
3. Hands-on interactive exercises
4. Group reflection upon the learned QI/PS skill
5. Completion of post-workshop survey

Most workshops were one hour in length with the exception of the RCA and process mapping workshops which required 1.5–2 h each.

### Curriculum development process

Ten student leaders with prior background in quality improvement and patient safety set out to design an innovative model for curriculum development through case-based experiential learning. Their prior background came from a combination of work experience, research experience, quality improvement and patient safety lectures, and supplemental coursework through the IHI Open School. An iterative process was used for content development for each workshop starting with a review of the literature [22–24]. Information from the review was synthesized and supplemented with IHI Open School online curriculum as well as prior didactic lecture notes. This led to the formulation of lesson plans, presentation slides, multimedia content, and handouts for each workshop.

Student leaders then brainstormed ideas for an interactive hands-on component for each workshop, with the assistance of both online resources and faculty. Student leaders met with subject matter expert faculty to comprehensively review the proposed curricula and receive feedback on areas for improvement prior to hosting the workshop for students. After the completion of each workshop, content was continuously improved by student leaders biannually based on participant feedback.

### Hands-on workshop components

**Fig. 1:**
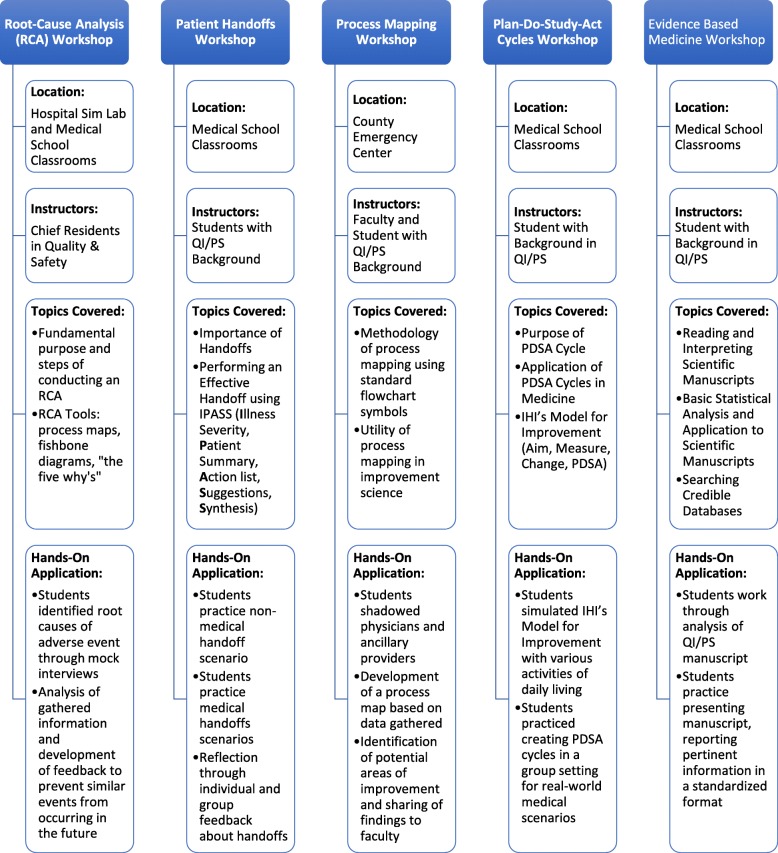
Structure and Content of Institute for Healthcare Improvement Workshops

Each workshop included a hands-on component in addition to a didactic session to engage learners in practically applying QI/PS skills in their careers (Fig. 1). The Patient Handoff workshop employed case-based learning for learners to practice effectively transitioning care between shifts and providers. The Process Mapping workshop provided learners with the opportunity to interview healthcare providers at a county hospital emergency department in order to observe, assess, and improve the efficiency of various clinical processes. The RCA workshop utilized case-based learning and role modeling to address prevention of adverse events and teach learners how to identify root cause of a medical error retrospectively. The PDSA cycle workshop gave learners the opportunity to simulate IHI’s Model for Improvement through real-world daily activities in addition to medical scenarios [25, 26]. The EBM workshop used case-based learning to teach learners how to use evidence-based medicine tools when analyzing, interpreting, and presenting manuscripts related to QI/PS scholarship.

### Workshop surveys

A survey development team consisting of IHI student leaders met annually to develop, adjust, and analyze survey questions for each workshop, in addition to analyzing data collected to date. Surveys were distributed before and after each workshop.

The first portion of each pre-workshop survey had three questions utilizing 5-point Likert scales to assess learners’ confidence in performing QI/PS skills, comfort level in teaching their peers the learned skill, and likelihood of pursuing quality improvement and patient safety throughout their careers. The middle of each pre-workshop survey assessed knowledge objectively through 1–3 multiple-choice and short answer questions. The end of each pre-workshop survey included questions regarding level of training, previous formal instruction regarding each learned skill, and the reason for interest in the workshop. The post-workshop survey mirrored the pre-workshop survey with the addition of 5-point Likert scales assessing usefulness of the workshop and effectiveness of the facilitator as well as qualitative free response and binary (yes/no) questions regarding strengths of the workshop, suggestions for improvement, and adequacy of time allotted. Qualitative data was collected primarily to receive feedback on the quality of the workshop and make improvements in the workshop content or structure for future sessions.

### Statistical Analysis

Statistical analysis was completed using either the McNemar test for correlated proportions or the non-parametric Wilcoxon signed-rank test for evaluating differences in paired ordinal data. Incomplete surveys with any non-free response questions left blank were excluded. All *p*-values are reported using one-tailed statistical testing since an educational intervention would be expected to have either no improvement or positive improvement in analysis of pre- and post-survey variables.

## Results

Complete data was collected from 88.5% of learners who attended the workshops (*n* = 185/209). A total of 13 workshops were offered with an average of 16 learners in attendance. Approximately 19.5% of learners had previously received formal instruction on the topic being taught at the workshop. A majority of those with prior exposure had taken a required Patient Safety course during their second year of medical school or participated in preclinical electives offered by the IHI Open School Chapter. A majority of learners were preclinical students in their first two years of professional school (Table 1).

**Table 1:** Institute for Healthcare Improvement Workshop Attendance

Workshop	Total # of Learners in Attendance	Medical Student (Year 1)	Medical Student (Year 2)	Medical Student (Year 3)	Medical Student (Year 4)	Other (Physician Assistant / Post-Graduate)	Unknown
Root-Cause Analysis	31	17	12	1	1	0	0
Patient Handoffs	67	41	23	0	0	1	2
Process Mapping	41	31	7	1	1	1	0
Plan-Do-Study-Act Cycles	39	32	5	0	0	1	1
EvidenceBased Medicine	31	12	9	0	0	0	10

### Attitudes

**Fig. 2:**
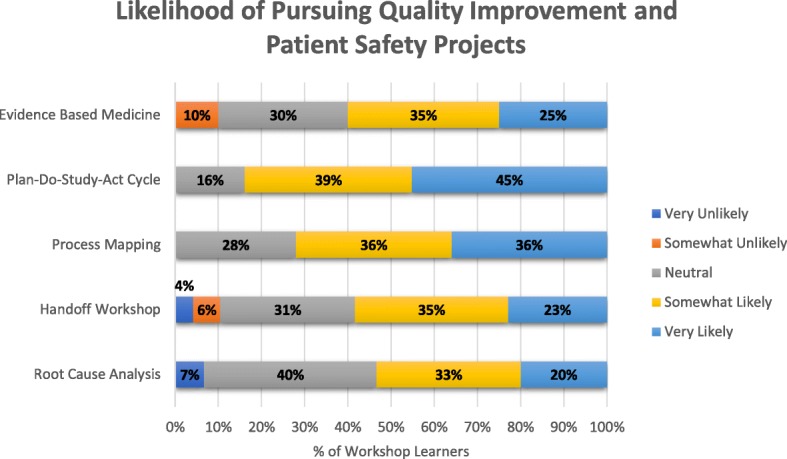
Post-Workshop Likelihood of Students Pursuing Quality Improvement and Patient Safety Projects throughout their Careers

Overall, 80.0% of learners believed that the workshops were useful. Moreover, after attending the workshops, learners were more likely to pursue QI/PS projects in their careers (mean pre/post difference 0.45, *p* < 0.0001, *n* = 139) than they were before attending. Post-workshop survey data indicated that over half of students attending each type of workshop were at least somewhat likely to pursue QI/PS projects (Fig. 2).

### Behaviors & Knowledge

Each workshop showed a statistically significant improvement in self-reported confidence to perform and comfort level to teach peers regarding each learned skill (Table 2). Across all workshops together, learners demonstrated a significant improvement in self-reported confidence to perform the learned QI/PS skill (mean pre/post difference 1.95, p < 0.0001, *n* = 185) and in self-reported comfort level to teach peers the QI/PS skill (mean pre/post difference 1.96, p < 0.0001, n = 139). Lastly, learners showed an increase objectively in knowledge at all workshops, with EBM, process mapping, and RCA workshops leading in percentage of improvement (Table 2).

**Table 2:** Change in Confidence, Comfort Teaching Colleagues, and Knowledge of Learned Skills Pre- and Post-Workshop

	Confidence		Comfort Teaching Colleagues		Knowledge		
Workshop	Mean Pre/Post Difference	*p-value (n)	Mean Pre/Post Difference	*p-value (n)	Pre-Workshop Proportion Correct	Post-Workshop Proportion Correct	**p-value (n)
Root-Cause Analysis	1.65	< 0.0001 (31)	1.60	0.0015 (15)	0.26	0.96	< 0.0001
Patient Handoffs	2.24	< 0.0001 (62)	2.29	< 0.0001 (48)	0.80	0.91	0.0625
Process Mapping	2.07	< 0.0001 (41)	2.40	< 0.0001 (25)	0.43	0.96	< 0.0001
Plan-Do-Study-Act Cycles	2.16	< 0.0001 (31)	1.94	< 0.0001 (31)	0.48	0.85	< 0.0001
Evidence Based Medicine	0.90	0.0018 (20)	0.95	0.0018 (20)	0.18	0.88	< 0.0002

*Calculated using one-tailed Wilcoxon signed-rank test

**Calculated using one-tailed McNemar test

### Length of workshop

Cumulative feedback revealed that 90.0% (*n* = 129) of workshop attendees believed that the time allotted for each workshop was adequate. Learners who felt that time was not adequately allotted provided qualitative feedback that included reducing didactic session time in favor of more hands-on session time for portions of the workshop.

### Facilitator evaluations

Facilitators for a majority of workshops were graded on their effectiveness utilizing a 5-point Likert scale. The mean score for facilitators from all workshops was 4.77 ± 0.47 (*n* = 113). Patient handoffs and PDSA cycle workshops had repeat facilitators.

### Qualitative feedback

When learners were asked about why they were interested in participating in the workshops, sample responses included the following: “to learn about system-based changes”, “to better understand workflow and patient experience”, “to develop innovative ways to improve health care delivery”, “see the practicality of hospital operations”, “to learn skills necessary to prevent medical errors that are costly to patients and the healthcare system”, and “to prepare for clinical rotations and residency.” Positive feedback from learners about workshops included the following: “interactive style of learning was much more engaging than traditional lecture style”, “good exposure to patient flow in the emergency room and application of real-life quality improvement methods”, “good audience participation”, and “this material should be taught to all students.” Suggestions for improvements from learners included the following: “smaller group sizes for discussion”, “provide handouts over material taught for students to take home”, “condense didactic time to add additional hands-on practice time”, “practice skills under time constraint”, and “include more video examples.”

### Total activity time

The total time spent on preparing, executing, reflecting, and improving each offering of the workshop varied based on topic. A plan-do-study-act model was used to improve workshops from one offering to the next. The estimated time commitment for student leaders to ‘plan’ and ‘do’ a workshop for the first time was approximately 8–12 h. The majority of this time was spent in the preparation phase (content and curriculum development). The ‘study’ and ‘act’ phases typically took 1–2 h, depending on the complexity of the specific workshop. The subsequent PDSA cycle required minimal time (less than 1–2 h) to ‘plan’ the workshop since the curriculum foundation had already been established. Including time spent at each workshop, faculty typically dedicated a total of 2–4 h of time for first-time workshop offerings and 1–2 h of time for repeat workshops offerings.

## Discussion

In this pilot study, we sought to assess the impact of an innovative model of medical education: a student-led initiative to educate health science students regarding basic principles and skills of QI/PS. We assessed learners’ attitude, behavior, and knowledge as well as their previous exposure to workshop topics. Feedback obtained from each workshop allowed us to continuously revise course content and improve upon the quality of our workshops for future attendees.

Several key lessons were learned throughout the completion of this pilot study. First, student-led skills workshops were found to effectively improve knowledge, attitudes, and behaviors of health science students towards QI/PS. Students had an increased likelihood of pursuing QI/PS projects throughout their careers after attending the skills workshops and perceived the workshops to be useful. The process mapping, PDSA cycle, and patient handoffs workshops had larger improvements in confidence to perform and comfort level to teach the learned skill, compared to the RCA and EBM workshops. This is likely due to both the complexity of the RCA and EBM material as well as a lower likelihood of immediate application of these skills in early medical training. Nonetheless, students demonstrated an ability to grasp these complex concepts as knowledge improved as a result of each workshop. Workshops with minimal improvement in knowledge, such as patient handoffs, had attendees with a relatively strong baseline knowledge of the material. Notably, however, knowledge of material may not necessarily be indicative of competency or confidence in performing the specific QI/PS skill.

Second, students expressed a genuine interest in learning QI/PS skills early in their medical training. Very few learners in attendance reported having any exposure to QI/PS in a real-world clinical or research setting. Common themes identified in reasons for attending workshops hosted by the IHI Open School chapter included career interest in QI/PS, preparation for rotations or residency training, the opportunity to learn about healthcare system processes, and lack of knowledge or hands-on exposure to QI/PS in medical training.

Third, students frequently expressed that the experiential, hands-on portion of each workshop was instrumental in developing competencies required to perform a learned QI/PS skill [27, 28]. Numerous studies have confirmed the benefit of this approach of bridging the gap between theoretical concepts and real-world application within clinical practice [19, 29, 30]. This type of teaching methodology likely intrigued students when they chose to attend the workshop. Qualitative feedback indicated that they were better able to understand the utility of the material after seeing its practical application in simulation activities.

Fourth, the time commitment required to design and implement the QI/PS workshops was reasonable from both a student and faculty perspective. Workshop attendees only dedicated 1–2 h of their time to learn a valuable QI/PS skill. Student leaders involved in designing or leading workshops also were able to effectively balance their coursework (including clinical rotations) with the time commitment required to design and implement a hands-on QI/PS curriculum. Since much of this initiative was student-driven and student-led, faculty also enjoyed having an advisory role without the burden of a large time commitment. Some faculty preferred communicating simply via email and providing feedback and edits to learning material developed by students. Others preferred meeting once or twice briefly for an hour or less prior to a workshop to review the content of the workshop and prepare the lesson plan. Repeat workshops with familiar faculty involved in a prior workshop administration often required minimal time commitment (usually less than thirty minutes) outside of the workshop itself. This type of system further propagates student leadership and achieves buy-in from faculty in busy academic centers that are interested in teaching QI/PS, but whom have limited time available for self-created content.

Lastly, this pilot study highlights the success of an innovative approach comprised of student-directed learning to fill educational gaps in QI/PS training [31]. Student leaders of the IHI chapter were able to design the curriculum for the skills workshops, and also take ownership of the delivery of each workshop. By receiving instruction from peers, workshop attendees likely felt a heightened sense of relevance towards the material being taught. Student leaders were also able to share personal experiences regarding the applicability and practicality of the material during their own medical training. This likely encouraged students to have raised personal awareness of trends within the healthcare system that ultimately affect the quality of care provided to patients.

This initiative had many facilitating and hindering factors that ultimately led to the described outcomes (Table 3). As more workshops were offered over time, numerous actions were taken to reduce hindering factors. For example, coordinating scheduling of workshops to allow both preclinical and clinical students to attend the workshops proved to be a significant barrier. To address this, student leaders reached out to the Internal Medicine clerkship leadership to assist in allowing clerkship students to receive protected time to step away from clinical duties in order to attend. Workshops were also purposely not scheduled near holiday or exam weeks. This type of planning not only raised attendance at each workshop, but also gave preclinical and clinical students the opportunity to learn from each other during the workshops. Additionally, faculty with experience in QI/PS were deliberately chosen from different specialties, when possible, to appeal to a wider audience within the student body. To reduce pressure from students’ competing interests, the IHI student group co-hosted workshops with other organizations at the medical school.

**Table 3:** Facilitating and Hindering Factors for Implementation of Quality Improvement and Patient Safety Workshops

Facilitating Factors	Hindering Factors
Large academic medical center with abundance of faculty with QI/PS experience	Competing interests and commitments of trainees (choosing between multiple lectures or academic organization events)
Funding for workshops to purchase any required materials and provide food for attendees	Schedule variation between preclinical students (basic sciences) and clinical students on rotations
Culture of safety within large academic medical center	Lack of awareness and familiarity regarding QI/PS terminology and skills
Engagement of student-leaders in Institute for Healthcare Improvement Open School Chapter	Access due to geographic location of workshop (especially for clinical students rotating at specific hospitals)
Interactive nature of workshops	Engagement of students with varying specialty interests (i.e. pediatrics, obstetrics/gynecology, internal medicine, surgery, etc.)
Opportunity for preclinical students to gain clinical exposure	Ability to accommodate large groups and maintain interactive nature of workshops
Opportunity for QI/PS faculty to get involved with medical school teaching	
Opportunity for faculty educational portfolio enhancement	

As an improvement-based organization, it was important for us to consider strategic changes to workshops based on feedback from post-workshop surveys. Workshop modifications included the following: (1) smaller discussion groups to increase individual participation, (2) availability of handouts with a lesson summary for students to take home, (3) addition of multimedia content to improve student engagement [32], (4) adjustment of workshop content based on perceived applicability and relevance, and (5) more time for hands-on practice of each QI/PS skill.

In summary, our data suggests that students understood the utility of learning QI/PS skills at such an early stage in their medical training and were receptive to teaching from peers in a hands-on interactive setting, as opposed to a formal, didactic lecture style of learning. Students are valuable participants within the healthcare system and are uniquely positioned to bring a fresh perspective to quality and safety and ultimately make an impact in the improvement of health care [33–35].

The strength of our study is derived from its prospective nature, with the availability of paired pre- and post-survey data to effectively determine the impact of a self-directed educational intervention for health sciences students. This study has the advantage of incorporating a modified version of the successful ‘flipped classroom’ methodology, by primarily focusing on active experiential learning with minimal didactic instruction [20, 21]. The pilot study and curriculum development itself was successfully created with limited resources and minimal faculty involvement. The initiative was openly accepted and welcomed by students, faculty, and educational administrators. This is evidenced not only by the voluntary nature of each workshop, but also the level of participation each semester. The study also speaks to the importance that faculty place on QI/PS topics as all faculty involved in the implementation of these workshops were recruited voluntarily.

There are several limitations to this study. First, the sample size of the study is limited to approximately 20% of all students enrolled in medical or allied health programs in a single institution within a given year. As such, the results may not be generalizable to all medical or allied health students due to the existence of selection bias within workshop participants, who voluntarily chose to attend and therefore may have had pre-existing interest in QI/PS. As hands-on interactive workshops do not widely exist, we hope others can utilize this methodology to teach topics in quality improvement and patient safety. Second, pre- and post-workshop questionnaires were initially developed for internal quality improvement purposes within the IHI Open School chapter and were not independently validated. Third, there is inevitable variability that exists between workshops based on topics taught at each session. For this reason, data was stratified by workshop topic. Multiple offerings of the same workshop varied slightly due to improvements made based on feedback from attendees or different workshop facilitators. However, the central premise of focus on hands-on interactive learning as opposed to didactic learning remained consistent amongst workshops. Lastly, data obtained by surveys was self-reported and short-term; a need exists for a more robust, objective evaluation of students’ competency in performing each QI/PS skill and of whether workshop participation induced any long-term changes in knowledge, attitudes, or behaviors related to QI/PS. There is a need for further research to determine both the longitudinal impact of teaching QI/PS skills to medical students early in their training and the most effective teaching strategy to encourage students to learn, retain, and apply QI/PS principles. At this time, the effect of early QI/PS educational interventions on patient outcomes, including morbidity and mortality, remains unclear.

## Conclusion

With limited number of faculty trained in QI/PS at many academic centers, the framework of this pilot study can be utilized to rapidly develop an interactive curriculum that targets educational gaps within existing QI/PS curricula. Our innovative methodology of self-directed peer-to-peer teaching has the potential to be feasibly replicated at other medical schools and residency programs, allowing trainees to have the opportunity to gain hands-on simulated training to sharpen their QI/PS skills within medical practice. This methodology not only helps facilitate the dissemination of essential QI/PS principles in an increasingly value-driven healthcare environment, but also cultivates the teaching skills of future physician leaders, all while addressing a clear shortcoming within our current medical education system.

## Data Availability

The datasets used and/or analyzed during the current study are available from the corresponding author upon reasonable request.

## Abbreviations

AAMC: Association of American Medical Colleges
EBM: evidence based medicine
IHI: Institute for Healthcare Improvement
PA: physician assistant
PDSA: plan-to-study-act
QI/PS: quality improvement and patient safety
RCA: root-cause analysis
WHO: World Health Organization
